# 

**Published:** 1895-11-30

**Authors:** 


					ABSOLUTELY PORET therefore BEST. Novembep, 30th, 1895.
CADBURY'S ^ f ? CADBURY'S
COCOA 'Ml IIHI /M COCOA
1 "Ihe standard of highest pur'.iy at present NO ALKALIES USED,
attainable."?Latieet.
No. 479. Vol. XIX.
Saturday,
Nov. 30th, 1895.
WITH EXTRA NURSING SUPPLEMENT. ??k? twopence.
m _ _ __ . _ _ __ . Per Annum, 10s. 6d? post free.
[Registered at tbe General Post Office as a Newspaper for Monthly Parts, 6d.: post free, 8d.
transmission in the United Kingdom and Abroad.] Ditto per Annum, 8s.6d., post free.

				

